# A Comprehensive Digital Workflow for Enhancing Dental Restorations in Severe Structural Wear

**DOI:** 10.3390/bioengineering13010077

**Published:** 2026-01-10

**Authors:** Abdulrahman Alshabib, Jake Berger, Edgar Garcia, Carlos A. Jurado, Guilherme Cabral, Adriano Baldotto, Hilton Riquieri, Mohammed Alrabiah, Franciele Floriani

**Affiliations:** 1 Department of Restorative Dental Science, College of Dentistry, King Saud University, Riyadh 11545, Saudi Arabia; 2 Private Practice, Lakewood Ranch, FL 34202, USA; 3 Department of Prosthodontics, The University of Iowa College of Dentistry, Iowa City, IA 52242, USA; 4 Division of Operative Dentistry, Department of General Dentistry, College of Dentistry, The University of Tennessee Health Science Center, Memphis, TN 38103, USA; 5 School of Dental Medicine, Ponce Health Sciences University, Ponce, PR 00732, USA; 6 Consultant Midwest Dental Arts Inc., Palm Bay, FL 32909, USA; 7 Department of Prosthetic Dental Science, College of Dentistry, King Saud University, Riyadh 11545, Saudi Arabia

**Keywords:** digital workflow, dentistry, scanners, wear

## Abstract

Patients with severe structural tooth wear present significant restorative challenges, including compromised oral function and the loss of essential anatomical landmarks such as marginal ridges, incisal edges, cusps, occlusal planes, and vertical dimension of occlusion (VDO). Successful management requires meticulous diagnosis, comprehensive treatment planning, and careful selection of restorative materials with appropriate biomechanical properties. Digital technologies have become integral to this process, particularly for enhancing diagnostic accuracy, material selection, and tooth preparation design within a fully digital workflow. This clinical case report illustrates a complete digital approach, beginning with an initial intraoral scan merged with a digital wax-up STL file featuring varying translucency dimensions to guide tooth preparation. This workflow enabled precise planning of tooth reduction, accurate assessment of available interocclusal space, and determination of material thickness requirements prior to irreversible procedures. Additionally, the integration of digital visualization improved patient communication, treatment predictability, and interdisciplinary collaboration. Overall, this case highlights the value of CAD/CAM technology in supporting complex oral rehabilitation for patients with advanced tooth wear, demonstrating its capacity to enhance efficiency, precision, and outcome quality in full-mouth zirconia ceramic restorations.

## 1. Introduction

Tooth wear is defined as the progressive loss of dental hard tissues caused by chemical and/or mechanical processes unrelated to bacterial activity. Clinically, it is characterized by smooth, polished, flat, or sharply contoured surfaces, most commonly affecting occlusal and incisal areas, and frequently associated with excessive attrition between opposing teeth [1]. Although tooth wear is a physiological phenomenon associated with aging, pathological forms may develop when the rate of tissue loss exceeds the tooth’s reparative capacity, resulting in functional, esthetic, and structural compromise [2].

The etiology of tooth wear is multifactorial and often involves a complex interaction between intrinsic and extrinsic factors. Parafunctional habits such as bruxism, temporomandibular disorders, psychological stress, and the use of medications related to anxiety or depression have been widely associated with increased mechanical wear [3]. In addition, chemical erosion caused by intrinsic acids (e.g., gastroesophageal reflux) or extrinsic dietary sources contributes significantly to enamel and dentin degradation. Abrasion related to oral hygiene practices and occupational factors may further accelerate the loss of occlusal and incisal tooth structure. Recent epidemiological studies have demonstrated a growing global prevalence of tooth wear across different age groups, suggesting that both its incidence and severity are likely to increase in the coming decades as predisposing factors become more prevalent [4,5,6].

Beyond its structural implications, severe tooth wear has a profound impact on patients’ quality of life. Advanced cases may lead to dentin hypersensitivity, compromised masticatory efficiency, phonetic alterations, and esthetic concerns, all of which can negatively affect emotional well-being and social and professional interactions [7]. From a clinical standpoint, these consequences underscore the importance of tooth wear as a significant and increasingly common condition requiring comprehensive and individualized management strategies.

The rehabilitation of patients with advanced tooth wear presents considerable restorative challenges, particularly when extensive loss of dental tissues is accompanied by alterations in the vertical dimension of occlusion (VDO) [8,9]. Restoring function and esthetics in such cases demands careful assessment of occlusal relationships, inter-arch space, facial proportions, and adaptive capacity of the stomatognathic system. Consequently, predictable treatment outcomes rely on meticulous diagnostic protocols and well-structured treatment planning that integrate etiological control, functional rehabilitation, esthetic considerations, and long-term preventive strategies [10,11,12]. A multidisciplinary and personalized approach is often required to mitigate deleterious factors, reestablish occlusal harmony, and ensure long-term stability of the rehabilitation.

In recent years, significant advancements in adhesive dentistry, restorative materials, and digital technologies have fundamentally transformed the management of severe tooth wear. These developments have enabled more conservative restorative approaches by preserving remaining tooth structure while enhancing the predictability and durability of treatment outcomes [13,14,15]. In particular, digital workflows have introduced new possibilities for diagnosis, planning, and execution of complex rehabilitations, facilitating a more precise and controlled restorative process.

Digital technologies enhance diagnostic accuracy by enabling detailed intraoral visualization, precise assessment of interocclusal space, and comprehensive analysis of occlusal relationships [16,17]. Intraoral scanning combined with digital planning tools allows clinicians to evaluate restorative requirements virtually, reducing uncertainties associated with conventional analog techniques [18,19,20,21,22,23]. Moreover, digital design platforms provide an opportunity for clinicians and patients to visualize proposed treatment outcomes prior to irreversible procedures, thereby improving communication, patient acceptance, and informed decision-making [24,25,26].

Following the digital design phase, restorations are fabricated using computer-aided manufacturing (CAM) technologies, which may be broadly categorized into additive manufacturing (a-CAM) and subtractive manufacturing (s-CAM) processes. Additive manufacturing techniques, including three-dimensional printing and sintering, have evolved rapidly and are increasingly applied in dentistry for the production of provisional restorations, surgical guides, diagnostic models, and, more recently, definitive prosthetic components [27]. A wide range of materials—such as printable polymers, metal-based systems, ceramics, and zirconia—can be processed using different additive technologies, including stereolithography (SLA), digital light projection (DLP), selective laser sintering (SLS), fused deposition modeling (FDM), photopolymer jetting, binder jetting, and computed axial lithography [27].

Among these, SLA and DLP currently represent the most widely adopted additive manufacturing techniques in clinical practice. SLA is particularly advantageous for applications requiring high dimensional accuracy and surface detail, such as provisional and definitive prostheses, surgical guides, and diagnostic templates. In contrast, DLP technology offers faster fabrication times and is often preferred for producing larger components where speed is prioritized over ultra-fine resolution [28]. As these technologies continue to evolve, their role in comprehensive restorative workflows is expected to expand further.

In parallel, subtractive manufacturing remains a cornerstone of digital dentistry, particularly for ceramic restorations fabricated through CAD/CAM systems. Ceramic materials used in s-CAM workflows can be broadly classified into glass-matrix ceramics, resin-matrix ceramics, and polycrystalline ceramics [28]. Resin-matrix ceramics consist of ceramic fillers embedded within a polymer matrix, combining favorable mechanical properties with improved machinability. Glass-matrix ceramics include feldspathic ceramics, leucite-reinforced materials, and lithium disilicate, the latter being widely used in full-mouth rehabilitations due to its favorable balance between strength, esthetics, and adhesive bonding potential [29]. Polycrystalline ceramics, predominantly zirconia-based systems, exhibit superior mechanical properties and are available in varying translucency levels depending on yttria content, including multilayered configurations designed to optimize esthetic outcomes [30,31].

Despite these technological advances, the application of fully digital workflows in the management of severe tooth wear remains challenging. Alterations in VDO, complex esthetic demands, and the learning curve associated with implementing new digital tools continue to pose limitations in daily clinical practice [32]. Although several clinical reports have described digitally driven full-mouth rehabilitations [33,34,35], the literature remains limited regarding how digital tools can be systematically employed to guide restorative material selection and enhance communication between clinicians and dental technicians throughout the treatment process.

Therefore, the purpose of this clinical case report is to illustrate the clinical application of a fully digital workflow for the rehabilitation of severe tooth wear. The proposed approach integrates intraoral scanning with a digitally designed diagnostic wax-up and a translucency-guided tooth preparation strategy to support restorative planning and material selection. Rather than introducing a novel digital concept, this report aims to demonstrate how existing digital tools can be systematically combined to assist clinical decision-making, enhance communication between the clinician and dental technician, and facilitate the management of complex full-mouth rehabilitations in a patient-specific context.

## 2. Materials and Methods

**Figure 1:**
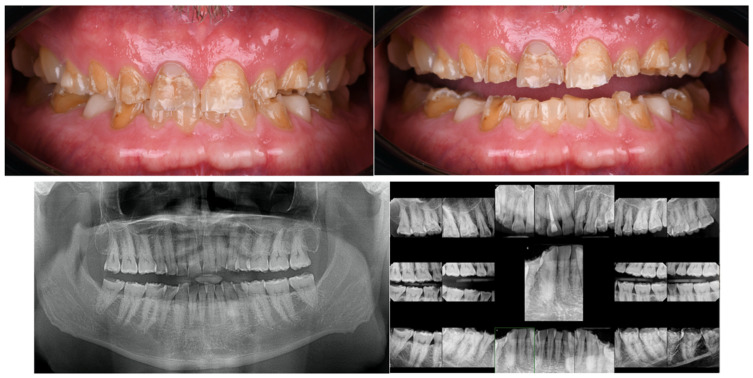
Intraoral photographs revealed a severely worn dentition with significant loss of tooth structure in the vestibular region and incisal/occlusal regions. A panoramic and full mouth series X-ray revealed severe tooth wear.

A 59-year-old Caucasian male presented to the clinic seeking improvement in occlusal function and anterior esthetics. His primary concerns included restoration of masticatory efficiency, enhancement of oral appearance, and recovery of self-confidence through comprehensive oral rehabilitation. The patient provided informed consent for all diagnostic and therapeutic procedures. Initial diagnostic records included standardized extraoral and intraoral photographs, full-mouth periapical radiographs, panoramic radiography, and digital impressions of both arches acquired using a chairside CAD/CAM intraoral scanner (Omnicam, CEREC; Dentsply Sirona, Charlotte, NC, USA). Clinical and radiographic evaluation revealed generalized severe tooth wear, fractures of previous restorations, and a collapsed vertical dimension of occlusion (VDO). Based on esthetic analysis, interocclusal space evaluation, and functional assessment, an increase of X mm in VDO was planned. The occlusal scheme selected was mutually protected occlusion, with anterior guidance established to disclude posterior teeth during excursive movements. An endodontic lesion was identified in tooth #9 and was treated prior to restorative procedures. Extensive loss of tooth structure was observed across multiple teeth, with pronounced vestibular erosion consistent with acid-related wear patterns (Figure 1). The proposed increase in VDO was first tested using provisional restorations, which were maintained for a period of X weeks/months. Acceptance of the new VDO and occlusal scheme was based on the absence of temporomandibular discomfort, muscle tenderness, phonetic impairment, or functional limitations, as well as patient-reported comfort and adaptation during the provisionalization phase.

**Figure 2:**
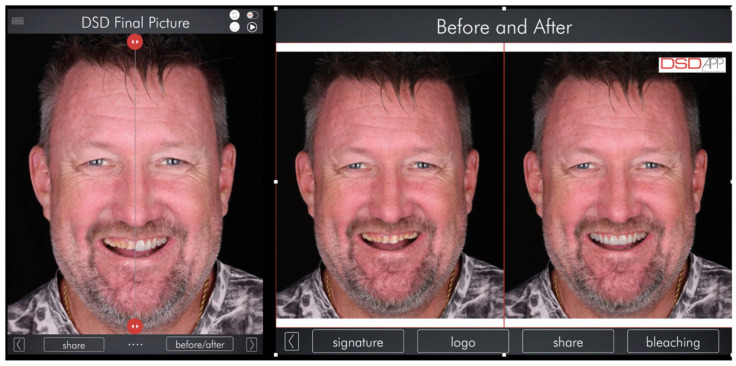
The virtual design software allowed for the assessment of potential changes to the patient’s facial appearance through superimposition and simulation of the planned oral rehabilitation.

**Figure 3:**
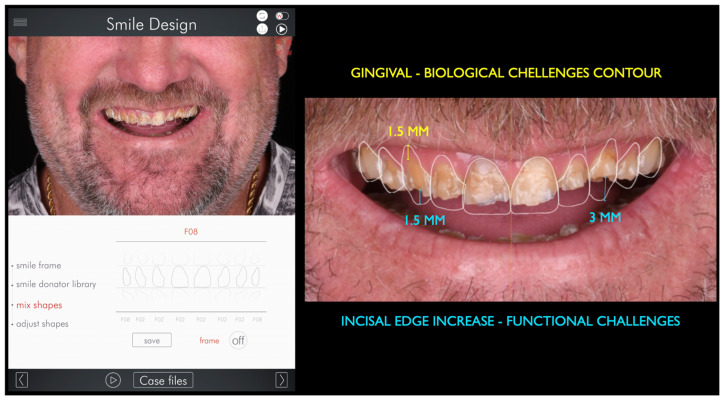
The DSD app virtual software was used to assess facial proportions and determine the optimal positions for both the gingival and incisal lines.

A comprehensive anamnesis and clinical examination identified multiple erosion- and wear-related risk factors. Preventive strategies were discussed with the patient, including dietary counseling and modifications in oral hygiene practices. Nocturnal bruxism was diagnosed based on a combination of clinical findings and patient-related data, including self-reporting, partner observations, signs of masticatory muscle fatigue and tenderness, pain assessment, polysomnography (PSG), and the American Academy of Sleep Medicine (AASM) self-reported questionnaire. Based on these findings, a multidisciplinary treatment plan was developed, integrating biological, functional, esthetic, and behavioral considerations. Given the severity of tooth wear, the presence of parafunctional activity, and the need for long-term durability, full-mouth rehabilitation using zirconia ceramic restorations was proposed and accepted by the patient. Secondary digital impressions of the maxillary and mandibular arches were obtained using the same intraoral scanner. The resulting STL files were imported into Digital Smile Design software (2023 version, DDS app, São Paulo, Brazil) for comprehensive esthetic and functional analysis. Facial analysis was performed using full-smile frontal photographs, with the horizontal reference plane established based on the interpupillary and intercommissural lines, and the facial midline defined by the glabella and philtrum. This digital planning phase supported the evaluation of dentogingival relationships, incisal edge positioning, smile line, and facial proportions, and allowed simulation of the anticipated post-rehabilitation facial appearance. The digital analysis also assisted in confirming the proposed increase in vertical dimension of occlusion and in guiding restorative material selection and tooth preparation planning (Figure 2 and Figure 3).

The Digital Smile Design (DSD) software enabled the assessment of potential changes in the patient’s facial appearance through image superimposition and digital simulation of the planned oral rehabilitation. Gingival zeniths and incisal edges were delineated during the digital planning phase to support periodontal and esthetic considerations in accordance with the digital smile design protocol (Figure 3).

**Figure 4:**
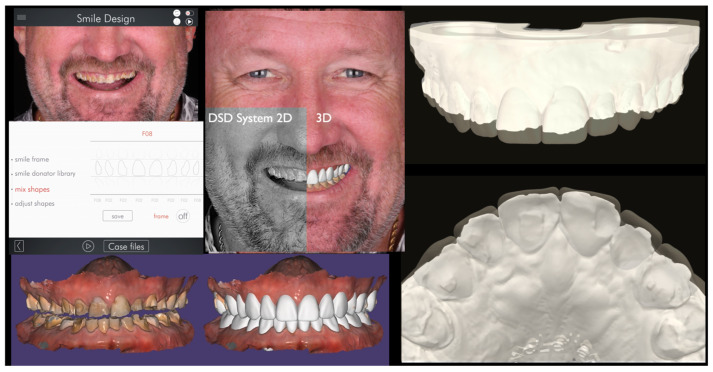
The DSD digital design was replicated within the Exocad software system. The 3D diagnostic wax-up STL file was integrated with the initial intraoral scan, incorporating various translucent dimensions to establish the preparation strategy and conduct an analysis of the minimum material thickness requirements.

Following esthetic planning, a three-dimensional diagnostic wax-up was developed using CAD software 2026 (DentalCAD, Rijeka, Exocad GmbH, Darmstadt, Germany), incorporating both functional and esthetic parameters. The wax-up STL file was merged with the original intraoral scans to generate a digital overlay, allowing precise mapping of required tooth reduction. Variable translucency parameters were incorporated into the digital design to visualize minimum material thickness requirements, ensuring a restorative thickness of approximately 1–2 mm for the selected zirconia material (Figure 4).

**Figure 5:**
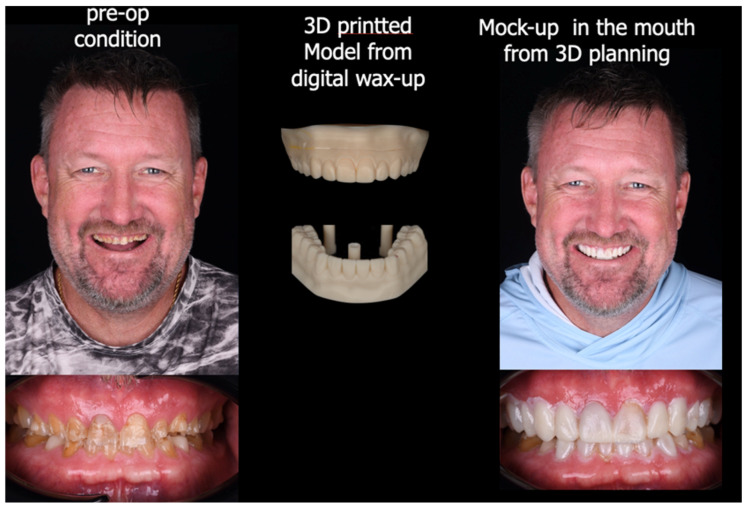
A functional mock-up was created using bis-acrylic self-cure temporary material to assess esthetics, functionality, occlusal vertical dimension, and phonetics.

Tooth preparation was guided by the digital wax-up and performed according to established geometric principles. Axial walls were prepared with a taper ranging from 8° to 15° in the cervical–occlusal direction, and all internal line angles were rounded to minimize stress concentration within the ceramic material. Occlusal reduction ranged from 1.5 to 2.0 mm, axial reduction was maintained at a minimum of 1.5 mm, and a chamfer margin design with approximately 1.0 mm thickness was employed. Preparations were performed conservatively to preserve enamel whenever possible, thereby optimizing adhesive bonding and biomechanical support. The diagnostic wax-up was exported for additive manufacturing using a 3D printer (Formlabs Inc., Berlin, Germany) to fabricate a physical model. A polyvinyl siloxane (PVS) index was produced and used to fabricate a functional mock-up with bis-acrylic self-curing temporary material (VOCO, Cuxhaven, Germany; shade A2). The mock-up allowed clinical evaluation of esthetics, phonetics, function, and the proposed VDO prior to definitive tooth preparation (Figure 5).

**Figure 6:**
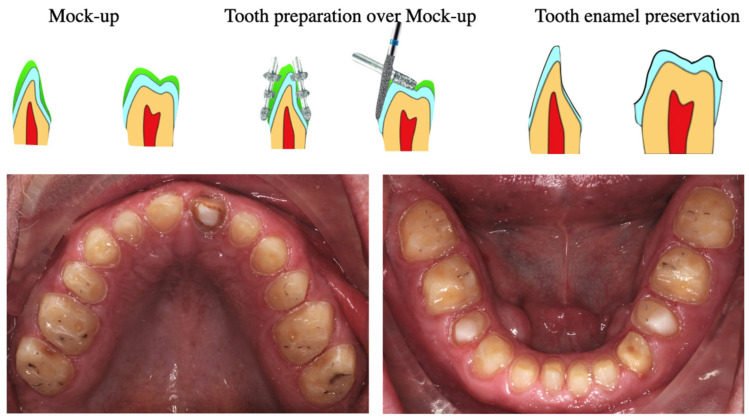
The preparation was carried out on top of the mock-up to preserve the tooth surface, ensuring long-term adhesive stability and better control of the support areas for the restorative material.

Clinical considerations at this stage included lip support, incisal edge positioning, and the identification of areas requiring additional preparation to achieve the requisite material thickness. Preparations were conducted judiciously to conserve tooth structure, especially enamel, to ensure optimal long-term adhesive bonding and a robust support for restorative materials. This conservative strategy allowed the selection of highly aesthetic materials with superior adhesive properties, enabling the use of monolithic zirconia restorations (Figure 6). The conservative preparation strategy and emphasis on enamel preservation applied in this case are in accordance with previously reported clinical approaches, including a clinical report on the restoration of anterior tooth defects using porcelain laminate veneers fabricated via the refractory technique, which illustrates similar minimally invasive principles through a different fabrication route.

**Figure 7:**
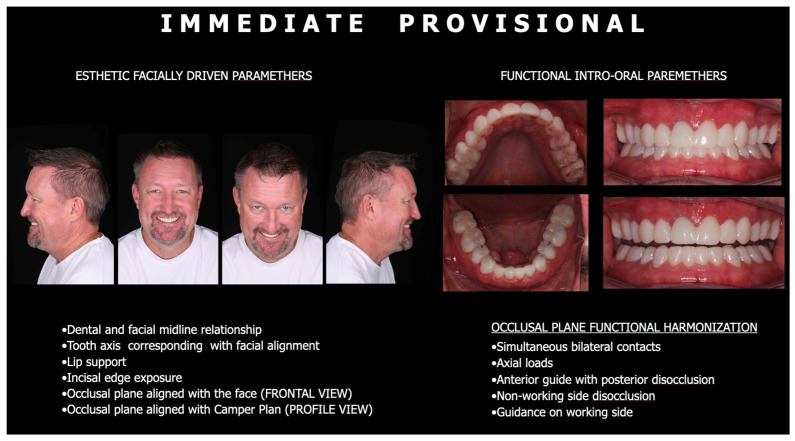
The provisionals were made to evaluate occlusal vertical dimension, occlusal contacts, esthetic and functional refinement of final full mouth oral rehabilitation.

The same polyvinyl siloxane (PVS) index was used to fabricate the provisional restorations, which were employed to evaluate occlusal contacts, stability of the vertical dimension of occlusion (VDO), esthetics, and functional comfort during an interim period (Figure 7). After intraoral adjustments and patient adaptation, a new digital impression was acquired to accurately transfer the established occlusal relationships to the definitive restorations.

After provisional restorations were adjusted intraorally, a new digital impression was captured to accurately replicate the occlusal dimension for the ceramic rehabilitation. This meticulous approach was instrumental in achieving predictable outcomes for the ceramic restorations.

Rubber dam isolation was provided from right first molar to left first molar with holding clamps #00 (Hu-Friedy, Chicago, IL, USA). Subsequently, the zirconia crowns were cleaned and subjected to airborne-particle abrasion with 50 µm aluminum oxide particles under controlled pressure, followed by ultrasonic cleaning in alcohol for 5 min.

The rationale for airborne-particle abrasion and the use of an MDP-containing adhesive system for bonding to zirconia is supported by prior laboratory evidence demonstrating that MDP-based light-cured veneer adhesive systems provide reliable shear bond strength to zirconia surfaces when appropriate surface preparation protocols are applied [29]. A zirconia-compatible primer containing 10-methacryloyloxydecyl dihydrogen phosphate (MDP) (Monobond Plus, Ivoclar Vivadent) was then applied to the internal surfaces of the restorations according to the manufacturer’s instructions and air dried. The tooth surfaces were conditioned by air abrasion using 20 µm aluminum oxide particles with water spray (AquaCare Aluminum Oxide Air Abrasion Power, London, UK). Enamel was selectively etched with 37% phosphoric acid gel (Ivoclar Vivadent, Schaan, Liechtenstein), followed by thorough rinsing and air drying. A universal adhesive system (BISCO Dental, Schaumburg, IL, USA) was applied to the prepared tooth surfaces and light-cured for 20 s using an LED curing unit (Valo, Ultradent, South Jordan, UT, USA).

**Figure 8:**
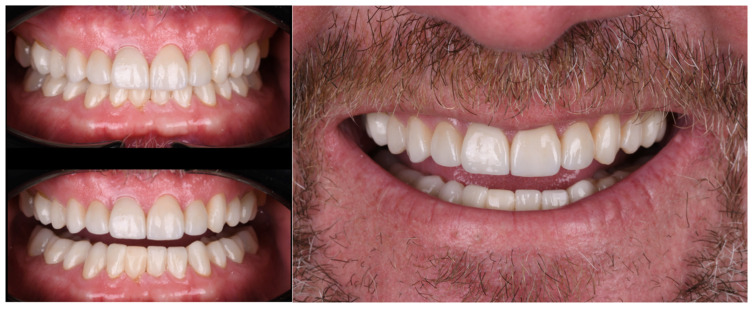
Intraoral aspects of final zirconia CAD/CAM full-mouth oral rehabilitation.

Finally, the crowns were cemented with resin cement light-shade (Ivoclar Vivadent, Schaan, Liechtenstein) starting for both central incisors, then both lateral incisors, then canines, premolars and molars. Excess of cement was meticulously removed and light-curing on the facial surface for 20 s, and floss was used to clean interproximal surfaces and another curing time of 20 s for each surface (incisal, mesial and distal) of the restorations. Oxygen inhibition layer was performed with the application of glycerine gel Liquid Strip (Ivoclar Vivadent, Schaan, Liechtenstein), and light-cured for 20 s. At this stage clamps were placed along the gingiva contours of the teeth to receive the restorations. Patient was provided with a full-mouth night guard to protect the restorations. Patient was fully satisfied with the shade, contours and occlusion of the new restorations (Figure 8).

**Figure 9:**
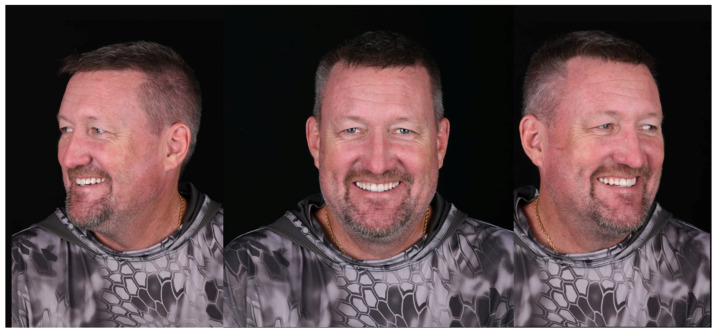
One-year follow-up of the CAD/CAM full-mouth oral rehabilitation.

At the one-year follow-up, the intraoral and facial aspects of the definitive milled zirconia ceramic restorations were evaluated, demonstrating successful integration and patient satisfaction (Figure 9). A mouth guard was delivered to protect the full mouth oral rehabilitation and provide a treatment for nocturnal bruxism.

## 3. Results

The fully digital workflow was applied to the planning and execution of a complex full-mouth rehabilitation in a patient presenting with severe generalized tooth wear. The integration of intraoral scanning data with a three-dimensional diagnostic wax-up supported the planning process, providing guidance for tooth preparation and assisting in the assessment of restorative space for monolithic zirconia restorations. The mock-up phase allowed clinical evaluation of esthetics, phonetics, functional parameters, and occlusal vertical dimension prior to definitive preparation.

Tooth reduction was performed in a conservative manner, aiming to preserve enamel and support adhesive procedures. Provisional restorations maintained occlusal relationships and patient comfort during the interim period. The definitive CAD/CAM-milled zirconia restorations demonstrated clinically acceptable marginal adaptation, anatomical contouring, occlusal relationships, and esthetic integration at delivery, requiring limited intraoral adjustment.

The adhesive cementation protocol resulted in stable seating of the restorations at delivery. The patient reported subjective improvement in masticatory function, speech, and esthetic appearance. At the one-year follow-up, clinical examination did not reveal signs of chipping, marginal discoloration, or debonding. Periodontal tissues appeared healthy, occlusal stability was maintained, and the patient expressed satisfaction with comfort, function, and appearance. A full-arch night guard was incorporated into the maintenance protocol to manage nocturnal bruxism.

## 4. Discussion

The present clinical case illustrates how an integrated digital workflow can support diagnostic planning and material selection in the rehabilitation of severe tooth wear. By merging intraoral scanning data with a diagnostic wax-up STL file designed with variable translucency parameters, restorative space requirements could be visualized, assisting tooth preparation planning prior to irreversible intervention. This approach facilitated a controlled and minimally invasive preparation strategy, aiming to preserve enamel whenever possible while accommodating restorative thickness requirements. Preservation of enamel is consistent with current recommendations for adhesive zirconia restorations, as enamel substrates have been associated with improved bonding reliability and favorable biomechanical behavior.

The digitally guided preparation assisted clinical decision-making and facilitated communication between the clinician and dental technician. In conventional analog workflows, interpretation of diagnostic wax-ups may be more subjective, potentially increasing the risk of over-preparation or insufficient restorative space. In this case, digital overlays of preparation guides and translucency maps allowed visual assessment of reduction requirements, supporting a balance between conservation of tooth structure and restorative design considerations. This approach may be particularly relevant in patients with severe tooth wear, where cumulative loss of dental tissues requires careful control of preparation geometry to minimize biological risks.

The literature supports the use of CAD/CAM-fabricated ceramic restorations for achieving clinically acceptable marginal adaptation and internal fit (Table 1 and Table 2) [36]. Improved manufacturing precision has been associated with reduced marginal discrepancies, which are factors related to cement stability and plaque control. When combined with conservative preparation designs, digitally milled restorations may contribute to favorable clinical behavior, especially in complex rehabilitations involving extensive occlusal reconstruction [37]. Statements regarding marginal and internal fit are supported by comparative laboratory studies evaluating ceramic laminate veneers fabricated using different CAD/CAM techniques, which indicate that such manufacturing methods are well documented in the literature. Accordingly, the present clinical report should be interpreted as an application of these established digital workflows rather than as a fundamentally new restorative protocol [38].

Recent investigations comparing additive and subtractive manufacturing techniques suggest that three-dimensionally printed zirconia restorations may achieve marginal accuracy comparable to that of milled and conventionally fabricated ceramics [38]. These findings indicate that additive manufacturing could represent a potential alternative in the future, particularly as materials and printing technologies continue to evolve. However, despite the promising results reported for additive techniques, subtractive CAD/CAM manufacturing remains the most established and clinically supported method for full-arch ceramic rehabilitations at present. Accordingly, material selection in the present case was guided by both emerging evidence and the need for well-documented mechanical performance in a patient presenting with parafunctional activity.

Material-related considerations are particularly relevant in the rehabilitation of severe tooth wear. When enamel preservation is achievable, lithium disilicate ceramics offer favorable esthetic characteristics and reliable adhesive potential. Nevertheless, in patients with bruxism or increased occlusal demands, high-strength zirconia restorations may represent a more suitable option in terms of mechanical resistance. Zirconia has been shown to exhibit higher fracture resistance than lithium disilicate, although its optical properties may vary depending on yttria content, microstructure, and sintering protocols [39]. Recent developments in translucent and multilayered zirconia systems have partially addressed previous esthetic limitations, supporting their clinical use in both anterior and posterior full-arch rehabilitations.

In contrast, lithium disilicate ceramics, while offering high esthetic potential, may be more sensitive to preparation design and adhesive protocols and may exhibit marginal changes during the crystallization process [40]. Although long-term clinical data for lithium disilicate restorations remain favorable, particularly for single-unit restorations, with reported five-year survival rates exceeding 96% [41], extrapolation of these outcomes to full-arch rehabilitations in patients with parafunctional habits should be interpreted with caution. In the present case, the selection of high-strength zirconia was therefore based on the patient’s functional demands, parafunctional activity, and the clinical objective of maintaining occlusal stability while achieving acceptable esthetic outcomes.

Beyond restorative material considerations, digital workflows may support interdisciplinary communication and patient engagement, both of which are important aspects in the management of severe tooth wear [42]. Digital visualization of occlusal schemes, esthetic modifications, and restorative dimensions can assist communication among clinicians, dental technicians, and patients prior to irreversible procedures [43]. This aspect may be particularly relevant in complex rehabilitations involving changes in the vertical dimension of occlusion, where patient adaptation and neuromuscular response must be carefully assessed.

Digital mock-ups and virtual simulations can facilitate shared decision-making by allowing patients to visualize proposed esthetic and functional changes. Such visualization has been associated with improved patient understanding and acceptance of comprehensive treatment plans [44,45]. In addition, digital previews may assist clinicians in evaluating phonetics, smile dynamics, and facial proportions, enabling refinement of treatment strategies before definitive execution.

An additional potential advantage of digital workflows is their contribution to long-term maintenance and monitoring of prosthetic rehabilitations [46]. The availability of archived digital records, including STL files, virtual articulations, and photographic documentation, provides a reference baseline for future assessment of wear progression, occlusal changes, or prosthetic complications [47]. In patients with bruxism-associated severe tooth wear, such documentation may assist in the design of protective appliances, evaluation of occlusal stability, and longitudinal monitoring of neuromuscular adaptation [48]. Moreover, the ability to reproduce restorations from stored digital designs may improve clinical efficiency should repairs or remakes become necessary [49].

Despite the favorable clinical outcome observed in this case, several limitations must be acknowledged. As a single-patient clinical report, the findings cannot be generalized to broader patient populations. In addition, the relatively short follow-up period limits conclusions regarding the long-term performance of the proposed digital workflow and material selection. Future prospective studies with larger sample sizes and longer follow-up periods are required to further assess the clinical behavior of fully digital approaches in the rehabilitation of severe tooth wear. Further research should also investigate the comparative clinical performance of milled versus additively manufactured ceramic restorations, as well as the refinement of digital planning tools to support restorative outcomes and longevity.

## 5. Conclusions

This clinical case illustrates the application of a fully digital workflow, starting from an initial intraoral scan merged with a digital diagnostic wax-up STL file with varying translucency levels. This approach supported the planning of tooth preparation, estimation of interocclusal space, and assessment of material thickness requirements. The individualized digital workflow assisted in treatment planning and material selection according to the specific demands of the case and resulted in restorations that were clinically acceptable in terms of function and esthetics.

## Figures and Tables

**Table 1:** Composition of milled resin composite.

Example of Commercial Name	Composition of Commercial Names	Advantages
Tetric CAD (Ivoclar, Schaan, Liechtenstein), Paradigm MZ100 (3M ESPE, St. Paul, MN, USA), Lava Ultimate (3M ESPE), Shofu Block HC (Shofu, Kyoto, Japan), Vita Enamic (Vita)	(1) Tetric CAD (Ivoclar, Schaan, Liechtenstein): Bis-GMA, Bis-EMA, TEGDMA, UDMA, barium aluminum silicate glass, silicon dioxide. Fillers by weight is 71.1%. (2) Paradigm MZ100 (3M ESPE): BisGMA, TEGDMA, ultrafine zirconia-silica ceramic particles. Fillers by weight is 85%. (3) Lava Ultimate (3M ESPE): Bis-GMA, Bis-EMA, TEGDMA, Silica and zirconia nanoparticles and nanoclusters. Fillers by weight is 80%. (4) Shofu Block HC (Shofu): TEGDMA, UDMA, Silica, fumed silica and zirconium silicate. Fillers by weight is 75%. (5) Vita Enamic (Vita): TEGDMA, UDMA, Feldspar ceramic enriched with aluminum oxide. Fillers by weight is 86%.	○ Indirect resin composites are polymerized extraorally overcoming some short-comings of direct composites such as polymerizations shrinkage, leach-able monomers, and enhanced mechanical properties. ○ Homogeneous material, industrially controlled, abrasion resistance, fracture toughness, hardness, easy in-mouth repair, smooth dissipation of masticatory loads, excellent compatibility with the opposing dentition, highly esthetic material. ○ Require minimal postprocessing steps, polishing, and possibly adding photopolymerizable stains for characterization to produce restorations such as veneers, inlays, onlays, and crowns.

**Table 2:** Composition of milled ceramics.

Ceramics	Example of Commercial Name	Minimal Material Thickness (mm) According to the Manufacture
Milled feldsphatic ceramics	Vitablocks Mark II (Vita Zahnfabrik, Bad Säckingen, Germany)	Inlay: 1.5 mm; onlay: 1.5–2.0 mm (occlusal). Anterior crown: 1.5 mm (incisal) and 1.0 mm (axial walls) Posterior crown: 1.5–2.0 mm (occlusal) and 1.0 mm (axial walls)
Milled Lithium disilicate ceramics	IPS e.max CAD (Ivoclar Vivadent, Schaan, Liechtenstein)	1. Crown: 1.0 mm (adhesive cementation); 1.5 mm Occlusal and 1.2 mm axial walls (self-adhesive cementation). 2. Inlay: 1.0 mm (occlusal). 3. Onlay: 1.0 mm (occlusal). 4. Veneer: 0.4–0.7 mm
Milled zirconia	(1) Zirconium oxide ceramic (ZrO_2_) VITA YZ (HT) (Vita Zahnfabrik, Bad Säckingen, Germany). (2) Zirconia-reinforced lithium silicate ceramic (ZLS) Celtra Duo (Dentsply Sirona Inc., Charlotte, NC, USA).	Zirconium Oxide Ceramic: Crown: 0.5 mm (occlusal) and 0.4 mm (axial walls) Bridge: 0.7 mm (occlusal) and 0.6 mm (axial walls) Zirconia-reinforced Lithium Silicate Ceramic: Crown: 1.5 mm (occlusal) and 10.0 mm (axial walls) Inlay: 1.5 mm (occlusal) Onlay: 1.5–2.0 mm (occlusal) Veneer: 1.0–1.5 mm (incisal) and 0.4–0.6 mm (axial walls)

## Data Availability

Data are contained within the article.

## References

[1] Cunha-Cruz J., Pashova H., Packard J.D., Zhou L., Hilton T.J., for Northwest PRECEDENT (2010). Tooth wear: Prevalence and associated factors in general practice patients. Community Dent. Oral Epidemiol..

[2] Sperber G.H. (2017). Dental Wear: Attrition, Erosion, and Abrasion-A Palaeo-Odontological Approach. Dent. J..

[3] Mehta S.B., Loomans B.A.C., van Sambeek R.M.F., Pereira-Cenci T., O’Toole S. (2023). Managing tooth wear with respect to quality of life: An evidence-based decision on when to intervene. Br. Dent. J..

[4] Yang J., Cai D., Wang F., He D., Ma L., Jin Y., Que K. (2016). Non-carious cervical lesions (NCCLs) in a random sampling community population and the association of NCCLs with occlusive wear. J. Oral Rehabil..

[5] Wetselaar P., Vermaire J.H., Visscher C.M., Lobbezoo F., Schuller A.A. (2016). The Prevalence of Tooth Wear in the Dutch Adult Population. Caries Res..

[6] Marró M.L., Aránguiz V., Ramirez V., Lussi A. (2020). Prevalence of erosive tooth wear in Chilean adults, 2016: A cross-sectional study. J. Oral Rehabil..

[7] Al-Khalifa K.S. (2020). The Prevalence of Tooth Wear in an Adult Population from the Eastern Province of Saudi Arabia. Clin. Cosmet. Investig. Dent..

[8] Afrashtehfar K.I., Manfredini D. (2013). Five things to know about bruxism. J. N. J. Dent. Assoc..

[9] Loomans B., Opdam N., Attin T., Bartlett D., Edelhoff D., Frankenberger R., Benic G., Ramseyer S., Wetselaar P., Sterenborg B. (2017). Severe Tooth Wear: European Consensus Statement on Management Guidelines. J. Adhes. Dent..

[10] Dallari G., Scalzo I., Rosati R.M., Sampaio C.S., Hirata R. (2021). Full-mouth adhesive rehabilitation of a severe case of erosion treated with v-shaped veneers. J. Esthet. Restor. Dent..

[11] Vailati F., Gruetter L., Belser U.C. (2013). Adhesively restored anterior maxillary dentitions affected by severe erosion: Up to 6-year results of a prospective clinical study. Eur. J. Esthet. Dent..

[12] Calamita M., Coachman C., Sesma N., Kois J. (2019). Occlusal vertical dimension: Treatment planning decisions and management considerations. Int. J. Esthet. Dent..

[13] Lanis A., Gallucci G., Pedrinaci I. (2023). Full mouth oral rehabilitation of a severely worn dentition based on a fully digital workflow. J. Esthet. Restor. Dent..

[14] Stawarczyk B., Meinen J., Wuersching S.N. (2024). Two-body wear of novel monolithic lithium-silicate ceramic materials and their corresponding different antagonists. J. Dent..

[15] Luna-Domínguez C.R., Luna-Domínguez J.H., Blatz M. (2023). Full-mouth rehabilitation in a completely digital workflow using partially adhesive monolithic zirconia restorations. J. Esthet. Restor. Dent..

[16] Ferrando-Cascales Á., Astudillo-Rubio D., Pascual-Moscardó A. (2020). A facially driven complete-mouth rehabilitation with ultrathin CAD-CAM composite resin veneers for a patient with severe tooth wear: A minimally invasive approach. J. Prosthet. Dent..

[17] Jurado C.A., Lee D., Cortes D., Kaleinikova Z., Hernandez A.I., Donato M.V., Tsujimoto A. (2023). Fracture Resistance of Chairside CAD/CAM Molar Crowns Fabricated with Different Lithium Disilicate Ceramic Materials. Int. J. Prosthodont..

[18] Stefanelli L.V., Franchina A., Pranno A., Pellegrino G., Ferri A., Pranno N., Di Carlo S., De Angelis F. (2021). Use of Intraoral Scanners for Full Dental Arches: Could Different Strategies or Overlapping Software Affect Accuracy?. Int. J. Environ. Res. Public Health.

[19] Fouda A.M., Atta O., Özcan M., Stawarczyk B., Glaum R., Bourauel C. (2023). An investigation on fatigue, fracture resistance, and color properties of aesthetic CAD/CAM monolithic ceramics. Clin. Oral Investig..

[20] Jurado C.A., Afrashtehfar K.I., Hyer J., Alhotan A. (2023). Effect of sintering on the translucency of CAD-CAM lithium disilicate restorations: A comparative in vitro study. J. Prosthodont..

[21] Alikhasi M., Yousefi P., Afrashtehfar K.I. (2022). Smile Design: Mechanical Considerations. Dent. Clin. N. Am..

[22] Passos L., de Vasconcellos A.B., Kanashiro L., Kina S. (2023). The natural CAD/CAM anterior implant single tooth restoration: A novel digital workflow. J. Esthet. Restor. Dent..

[23] Al-Wahadni A., Abu Rashed B.O., Al-Fodeh R., Tabanjah A., Hatamleh M. (2023). Marginal and Internal Gaps, Surface Roughness and Fracture Resistance of Provisional Crowns Fabricated with 3D Printing and Milling Systems. Oper. Dent..

[24] Young Kim R.J., Cho S.M., Jung W.S., Park J.M. (2024). Trueness and surface characteristics of 3-dimensional printed casts made with different technologies. J. Prosthet. Dent..

[25] Garaicoa J., Jurado C.A., Afrashtehfar K.I., Alhotan A., Fischer N.G. (2023). Digital Full-Mouth Reconstruction Assisted by Facial and Intraoral Scanners: A Case Report and Technique Description. Appl. Sci..

[26] Azpiazu-Flores F.X., Lee D.J., Jurado C.A., Nurrohman H. (2023). 3D-Printed Overlay Template for Diagnosis and Planning Complete Arch Implant Prostheses. Healthcare.

[27] Khorsandi D., Fahimipour A., Abasian P., Saber S.S., Seyedi M., Ghanavati S., Ahmad A., De Stephanis A.A., Taghavinezhaddilami F., Leonova A. (2021). 3D and 4D printing in dentistry and maxillofacial surgery: Printing techniques, materials, and applications. Acta Biomater..

[28] Gracis S., Thompson V.P., Ferencz J.L., Silva N.R., Bonfante E.A. (2015). A new classification system for all-ceramic and ceramic-like restorative materials. Int. J. Prosthodont..

[29] Lupu I.C., Tatarciuc M.S., Vitalariu A.M., Bobu L., Diaconu D.A., Vasluianu R.I., Stamatin O., Cretu C.I., Dima A.M. (2025). Bonding Strategies for Zirconia Fixed Restorations: A Scoping Review of Surface Treatments, Cementation Protocols, and Long-Term Durability. Biomimetics.

[30] Al-Johani H., Haider J., Silikas N., Satterthwaite J. (2024). Effect of repeated firing on the topographical, optical, and mechanical properties of fully crystallized lithium silicate-based ceramics. J. Prosthet. Dent..

[31] Güth J.F., Magne P. (2016). Optical integration of CAD/CAM materials. Int. J. Esthet. Dent..

[32] Segundo Â.R.T.C., Saraiva S., de Castro C., Sesma N., Bohner L., Andretti F.L., Coachman C. (2023). CAD-CAM natural restorations-Reproducing nature using a digital workflow. J. Esthet. Restor. Dent..

[33] Ates S.M., Yesil Duymus Z. (2016). Influence of Tooth Preparation Design on Fitting Accuracy of CAD-CAM Based Restorations. J. Esthet. Restor. Dent..

[34] Delavy J., Lopez C., Franchini L., Rocca G.T., Saratti C.M. (2024). Myocentric relation in an additive esthetic rehabilitation within a fully digital workflow. Int. J. Esthet. Dent..

[35] Badalotti G., Lenz U., Balen P.L., de Oliveira G.R., Bacchi A. (2023). A conservative full-mouth ceramic rehabilitation for a severely worn dentition. Int. J. Esthet. Dent..

[36] Sailer I., Makarov N.A., Thoma D.S., Zwahlen M., Pjetursson B.E. (2015). All-ceramic or metal-ceramic tooth-supported fixed dental prostheses (FDPs)? A systematic review of the survival and complication rates. Part I: Single crowns (SCs). Dent. Mater..

[37] Cervino G., Fiorillo L., Arzukanyan A.V., Spagnuolo G., Cicciù M. (2019). Dental Restorative Digital Workflow: Digital Smile Design from Aesthetic to Function. Dent. J..

[38] Anh N.V., Ngoc V.T.N., Son T.M., Hai H.V., Tra N.T. (2025). Comparison of the Marginal and Internal Fit of Ceramic Laminate Veneers Fabricated with Four Different CAM Techniques. Int. J. Prosthodont..

[39] Shembesh M., Ali A., Finkelman M., Weber H.P., Zandparsa R. (2017). An In Vitro Comparison of the Marginal Adaptation Accuracy of CAD/CAM Restorations Using Different Impression Systems. J. Prosthodont..

[40] Gold S.A., Ferracane J.L., da Costa J. (2018). Effect of Crystallization Firing on Marginal Gap of CAD/CAM Fabricated Lithium Disilicate Crowns. J. Prosthodont..

[41] Rokhshad R., Tehrani A.M., Nahidi R., Zarbakhsh A. (2024). Fit of removable partial denture frameworks fabricated from 3D printed patterns versus the conventional method: An in vitro comparison. J. Prosthet. Dent..

[42] Soni M., Soni P., Soni P., Chokhandre S., Moni M., Gupta S. (2025). The Role of Digital Workflow in Customizing the Prosthetic Solutions: A Literature Review. J. Pharm. Bioallied Sci..

[43] Fasbinder D.J. (2010). Digital dentistry: Innovation for restorative treatment. Compend. Contin. Educ. Dent..

[44] Brawek P.K., Wolfart S., Endres L., Kirsten A., Reich S. (2013). The clinical accuracy of single crowns exclusively fabricated by digital workflow--the comparison of two systems. Clin. Oral Investig..

[45] Zeltner M., Sailer I., Mühlemann S., Özcan M., Hämmerle C.H., Benic G.I. (2017). Randomized controlled within-subject evaluation of digital and conventional workflows for the fabrication of lithium disilicate single crowns. Part III: Marginal and internal fit. J. Prosthet. Dent..

[46] Benic G.I., Sailer I., Zeltner M., Gütermann J.N., Özcan M., Mühlemann S. (2019). Randomized controlled clinical trial of digital and conventional workflows for the fabrication of zirconia-ceramic fixed partial dentures. Part III: Marginal and internal fit. J. Prosthet. Dent..

[47] Sailer I., Mühlemann S., Fehmer V., Hämmerle C.H.F., Benic G.I. (2019). Randomized controlled clinical trial of digital and conventional workflows for the fabrication of zirconia-ceramic fixed partial dentures. Part I: Time efficiency of complete-arch digital scans versus conventional impressions. J. Prosthet. Dent..

[48] Tabesh M., Nejatidanesh F., Savabi G., Davoudi A., Savabi O., Mirmohammadi H. (2021). Marginal adaptation of zirconia complete-coverage fixed dental restorations made from digital scans or conventional impressions: A systematic review and meta-analysis. J. Prosthet. Dent..

[49] Rojas-Rueda S., Floriani F., Abuhammoud S., Mohammed A., Afrashtehfar K.I., Jurado C.A. (2025). CAD-CAM Complete Dentures Manufactured Using Additive and Subtractive Manufacturing Techniques: A Feasible Clinical Approach for Managing Geriatric Patients With Advanced Residual Ridge Resorption. Case Rep. Dent..

